# Transcriptome Analysis of Low-Temperature-Induced Breaking of Garlic Aerial Bulb Dormancy

**DOI:** 10.1155/2019/9140572

**Published:** 2019-08-07

**Authors:** Yuhui Dong, Mengjiao Guan, Lixia Wang, Lei Yuan, Xiudong Sun, Shiqi Liu

**Affiliations:** State Key Laboratory of Crop Biology, Shandong Garlic Engineering Research Center, College of Horticulture Science and Engineering, Shandong Agricultural University, No. 61, Daizong Road, Tai'an, Shandong Province 271000, China

## Abstract

The long history of asexual reproduction of garlic using garlic cloves has resulted in virus accumulation and genetic depression. Propagation of garlic seedlings by tissue culture can both eliminate viruses and improve breeding efficiency. Aerial bulbs are the first-choice materials for breeding virus-free garlic seedlings under external conditions, but they show dormancy just like garlic bulbs. However, low temperatures can quickly break dormancy. In this research, we used a high-throughput sequencing method to sequence aerial bulbs during dormancy and after low-temperature-induced breaking of dormancy to screen out the key differentially expressed genes (DEGs) associated with low temperature and to provide a theoretical basis for exploring the molecular mechanism of low-temperature-induced breaking of aerial bulb dormancy. The high-throughput transcriptome sequencing results showed that 6,675 DEGs were upregulated and 36,596 DEGs were downregulated in the aerial bulbs given low-temperature treatment. Then, 19,507 DEGs were assigned KEGG annotations, among which most DEGs were annotated to the metabolism pathway (11,817 genes, accounting for 60.58%), followed by the genetic information processing pathway (4,521 genes, accounting for 23.18%). The DEGs were mostly concentrated in pathways such as protein processing in the endoplasmic reticulum, plant-pathogen interaction, plant hormone signal transduction, and ribosome biogenesis in eukaryotes, with significant enrichment. The key DEGs related to calcium signaling, hormonal signaling, and transcription factors were screened out, including *CaM*, *CDPK*, and *CML* in accessory pathways of calcium signaling; *GA20ox*, *GAI1*, and *GA2ox* in accessory pathways of hormonal signaling; and transcription factor genes such as *MYB*, *AP2/ERF*, *bHLH*, *MADS*, and *bZIP*. qRT-PCR verification results were consistent with the sequencing results, indicating that the transcriptome sequencing data were accurate and reliable. Our results provide a theoretical basis for breaking the dormancy of aerial bulbs with low-temperature treatment to produce virus-free seedlings and increase the output and quality of garlic.

## 1. Introduction

Garlic (*Allium sativum* L.), an annual or biennial herb in the lily family, has a long history of cultivation and originated in Central Asia. The edible parts of garlic are the bulbs, sprouts, and young shoots. Garlic, as one of the most popular vegetables, can also be used to produce green garlic and blanched garlic leaves. Garlic has high nutritional value, strong bactericidal activity, and cancer prevention, detoxification, intestinal cleaning, blood sugar reduction, and cardiovascular and cerebrovascular disease prevention activities [1]. However, the long history of asexual reproduction of garlic through garlic cloves has resulted in accumulation of viruses, genetic depression, smaller garlic heads, and a sharp decrease in output and quality. Additionally, the low coefficient of asexual reproduction seriously limits the development of garlic production. However, the application of tissue culture techniques can solve the above problems and enable the breeding of virus-free garlic seedlings. Aerial bulbs grow quickly from bulbils inside the receptacles of young garlic shoots and carry very few viruses and are characterized by a small volume, large quantity, high germination percentage, uniform germination, genetic stability, and a high likelihood of forming independent test-tube plantlets. They are the first-choice materials for the breeding of virus-free seedlings under external conditions and can also improve the utilization of young garlic shoots. Research shows that seedlings induced from garlic bulbs have a low seedling rate and abnormal shoot growth, but bulbs released from dormancy boast a high seedling rate and rapid growth. Aerial bulbs can go dormant right after being picked. Thus, if they are used to induce virus-free seedlings, their dormancy must first be broken. Research has shown that low temperatures can be used to quickly break the dormancy of aerial bulbs, which can produce remarkable breaking effects.

The transcriptome contains all the RNAs transcribed in an organism or cell in a particular state, including mRNA and noncoding RNA. Transcriptome sequencing has gradually become the foundation for studies on a genetic structure and function. Transcriptome resequencing data can help clarify many biological processes such as gene expression [2], elucidate gene expression profiles and gene expression control, identify biomarkers in tissues, discover genes, determine gene contents, and isolate conserved homologous genes through molecular systematics after experimental treatment or diseases [3–5]. Therefore, it is possible to identify many candidate functional genes in a research material through transcriptome sequencing technology. High-throughput sequencing is a quick and reliable method of transcriptome sequencing. In many studies, *de novo* assembly has been used to analyze the transcriptomes of nonmodel organisms with no reference genome [6], such as sweet potato, alfalfa, and tea tree [7, 8].

Plant dormancy and the breaking of dormancy are controlled by many genes and also affected by environmental and internal factors (e.g., hormones, sugars, and nitrogen-containing compounds) [9, 10]. Calcium is one of the mineral elements necessary for plant growth and is an important second messenger in cell signal transduction, participating in many vital processes in plants such as cell wall formation, membrane structure stabilization, regulation of enzymatic activity, and defense against diseases. In recent years, many experiments have shown that Ca^2+^ is also closely related to the control of dormancy. The breaking of plant dormancy requires the coordination and interaction of various hormones [11]. Abscisic acid (ABA) is a positive regulatory factor for inducing plant dormancy and a negative regulatory factor for plant germination. In many species, endogenous ABA is involved in the induction and maintenance of plant dormancy [12–14]. Garlic in a state of dormancy has a higher content of ABA. Gibberellins (GAs) can release dormancy, promote germination, and antagonize ABA. Transcription factors are protein molecules that can specifically react with *cis*-acting elements in the promoter regions of genes to control the specific spatiotemporal expression and expression levels of target genes. Transcription factors play a key role in plant germination [15].

In this research, a high-throughput sequencing platform was used to perform transcriptome sequencing and sequence analysis of aerial bulbs during dormancy and after low-temperature-induced dormancy breaking. Bioinformatics methods were used to conduct functional annotation and classification of differentially expressed genes (DEGs), preliminarily screen out the genes related to the breaking of low-temperature-induced dormancy in garlic aerial bulbs, and investigate the relationship between DEGs and the breaking of aerial bulb dormancy. These results could provide a theoretical basis for exploring the molecular mechanism of low-temperature-induced dormancy breaking in aerial bulbs and for the breeding of virus-free seedlings from garlic aerial bulbs.

## 2. Materials and Methods

### 2.1. Plant Materials

The experimental materials were aerial bulbs that were removed from buds of excised garlic stems. Some aerial bulbs (JXLT) were stored at 4°C for 3 weeks, after which plant culture and transcriptome sampling were conducted. The controls were aerial bulbs kept at room temperature (25 ± 2°C) (JXD). For transcriptome analysis, the aerial bulbs were quickly frozen with liquid nitrogen and kept at −80°C before use.

### 2.2. Culture of Aerial Bulbs

After disinfection, JXLT and JXD aerial bulbs were inoculated onto shoot induction medium and kept under illumination of 2,600 lx for 16 h d^−1^ at room temperature (25 ± 2°C). The bulbs were observed regularly for germination, and induction rates were recorded.

### 2.3. Measurement of Hormone Contents

Endogenous hormone contents of aerial bulbs were determined by high-performance liquid chromatography (HPLC).

Samples (0.1 g) were ground and soaked overnight with 1 mL 80% methanol at 4°C. After centrifugation for 10 minutes at 8,000 g, the residue was leached with 0.5 mL 80% methanol for two hours, and the supernatant was taken after centrifugation. The supernatant was dried by 40°C nitrogen gas and decolorized three times by 0.5 mL petroleum ether. The pH value of the lower water phase was adjusted to 2.8 with 1 mol/L citric acid solution and extracted three times with ethyl acetate. The organic phase is dried with nitrogen, and the compatibility of the mobile phase is up to 0.5 mL. The appropriate solution is filtered with a needle filter in a sample bottle with an inner liner for testing. The mobile phase passes through the column, and we begin to sample when the baseline is stable.

### 2.4. Total RNA Isolation

A TRIzol kit (Invitrogen, Carlsbad, CA, USA) was used to extract total RNA from aerial bulbs. The RNA was then incubated with 10 units of DNase I (Takara, Dalian, China) for 30 min at 37°C to remove genomic DNA. An Agilent 2100 Bioanalyzer (Agilent RNA 6000 Nano Kit) was used to assess the quality of the total RNA.

### 2.5. cDNA Library Construction and Transcriptome Sequencing

An OligoTex (reverse transcription) mRNA kit (Qiagen) was used to purify the mRNA containing poly(A) from the total RNA samples. An RNA lysis kit (Ambion) was then used to break down the mRNA into small fragments. With these small fragments as templates, we used random hexamer primers and reverse transcriptase (Invitrogen) to synthesize the first-strand cDNA and DNA polymerase I and RNase H to synthesize the second-strand cDNA. We next used a QIAquick PCR Extraction Kit (Qiagen) to purify the cDNA fragments and EB buffer solution to perform end joining and addition of poly(A). Then, we connected the short fragments with sequencing adaptors and performed PCR purification and product amplification to create a final cDNA library. A BGISEQ-500 instrument was used for high-throughput sequencing of the cDNA libraries. We filtered the original reads to obtain clean reads. We used Trinity [16] to assemble the clean reads and Tgicl [17] to perform clustering and eliminate redundant data in the assembled transcripts to obtain Unigenes.

### 2.6. Differential Expression Analysis, DEG Functional Annotation, and Enrichment Analysis

We used the DEGseq method to detect DEGs from JXD/JXLT [18]. We used the Blastx software [19] to annotate the DEGs in the NR and KEGG databases and then used the Blast2GO software [20] to obtain GO annotations for the DEGs from the NR annotations. We used the WEGO software [21] to examine the category-based statistics of DEG functions and also used the phyper function in the R software to conduct enrichment analysis.

### 2.7. Validation by qRT-PCR

According to the annotation results, we screened out DEGs that play key regulatory roles and selected nine genes among them to design specific primers and verify the sequencing results by real-time fluorescence quantitative PCR. The primers were synthesized by Tsingke Biological Technology Co. Ltd.

## 3. Results

### 3.1. Studies on the Breaking Effect of Low Temperature on Aerial Bulb Dormancy

**Figure 1:**
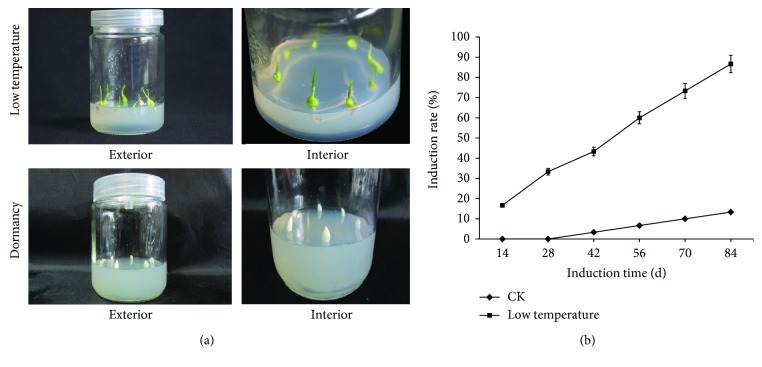
Breaking effect of low-temperature treatment on garlic aerial bulb dormancy. (a) Comparison of germination between aerial bulbs treated and untreated with low temperature after inoculation. (b) Schematic diagram of the germination induction rate of aerial bulbs treated with low temperature.

Low-temperature treatment produced a remarkable breaking effect on the dormancy of garlic aerial bulbs. The aerial bulbs treated with low temperature quickly germinated after inoculation, while the untreated aerial bulbs remained dormant after inoculation and did not germinate (Figure 1(a)). As the culture period was prolonged, the aerial bulbs treated with low temperature showed a significantly higher plantlet induction rate, while for the untreated aerial bulbs, the induction rate was zero at the early stage and then gradually increased but was still much lower than that of the treated aerial bulbs (Figure 1(b)).

### 3.2. RNA-Seq and *De Novo* Assembly

**Figure 2:**
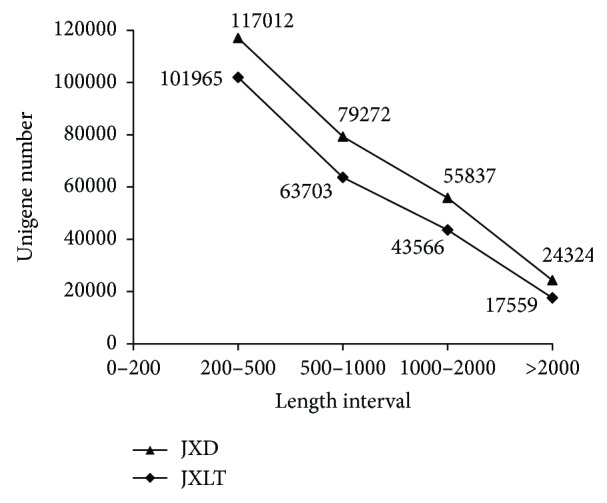
Quantitative distribution of Unigene length in JXD and JXLT.

We took the original image data from the sequencing of total RNA extracted from dormant aerial bulbs and bulbs after low-temperature-induced dormancy breaking and converted them into sequence data (raw reads) via base calling. After assembly and elimination of redundant data, we obtained 197.39 Mb of clean reads from dormant aerial bulbs and 200.15 Mb from aerial bulbs after low-temperature-induced breaking of dormancy. The total amounts of nucleobases obtained from the clean reads were 19.74 and 20.01 Gb, respectively (Table 1). We used Trinity to assemble clean reads and Tgicl to perform clustering and elimination of redundant data for the transcripts to obtain Unigenes. We obtained 276,445 Unigenes for dormant aerial bulbs (JXD) and 226,793 Unigenes for aerial bulbs after low-temperature-induced breaking of dormancy (JXLT). Most Unigenes were 200–500 bp in length; JXD contained 117,012 Unigenes in this length range while JXLT contained 101,965. Additionally, JXD contained 79,272 Unigenes of 500–1000 bp while JXLT contained 63,703. Furthermore, JXD contained 24,324 Unigenes over 2,000 bp while JXLT contained 17,559 (Figure 2).

### 3.3. Detection of Differentially Expressed Genes between JXD and JXLT

**Figure 3:**
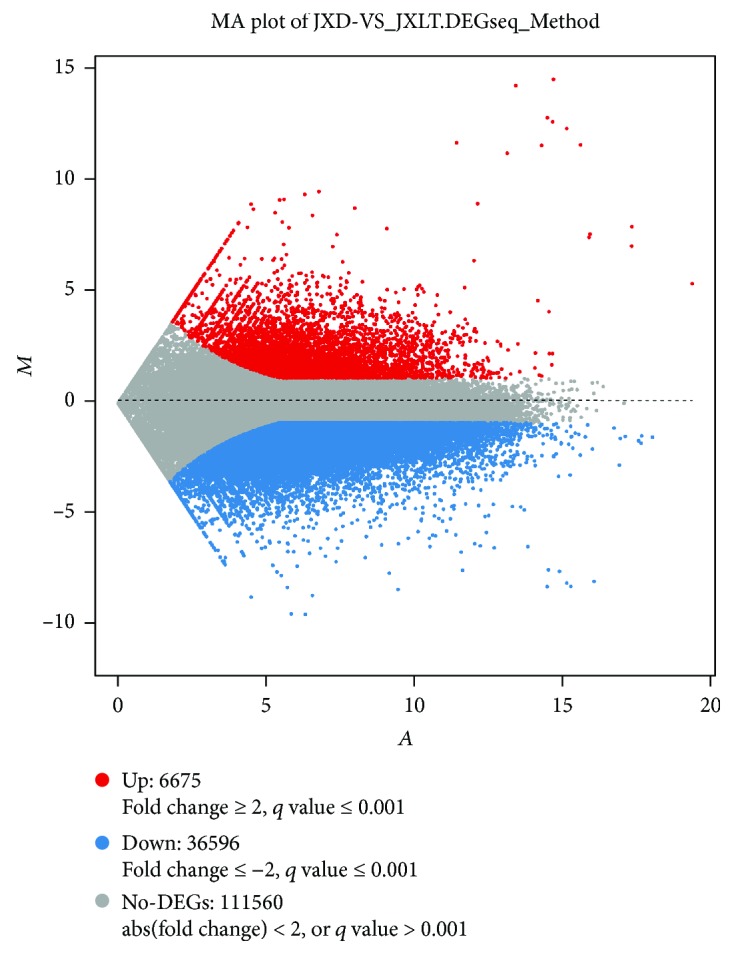
MA plot of DEGs from JXD/JXLT. *X*-axis represents value *A* (log2-transformed mean expression level). *Y*-axis represents value *M* (log2-transformed fold change).

Based on the gene expression levels of the samples, we used the DEGseq algorithm to detect DEGs. We also used a MA plot to show the distribution of DEGs from JXD/JXLT (Figure 3). A total of 43,271 DEGs were detected. Compared with dormant aerial bulbs, the aerial bulbs treated with low temperature contained 6,675 upregulated DEGs, accounting for 15.43%, and 36,596 downregulated DEGs, accounting for 84.57%. The number of downregulated DEGs was much greater than that of upregulated DEGs.

### 3.4. GO Functional Analysis of Differentially Expressed Genes

**Figure 4:**
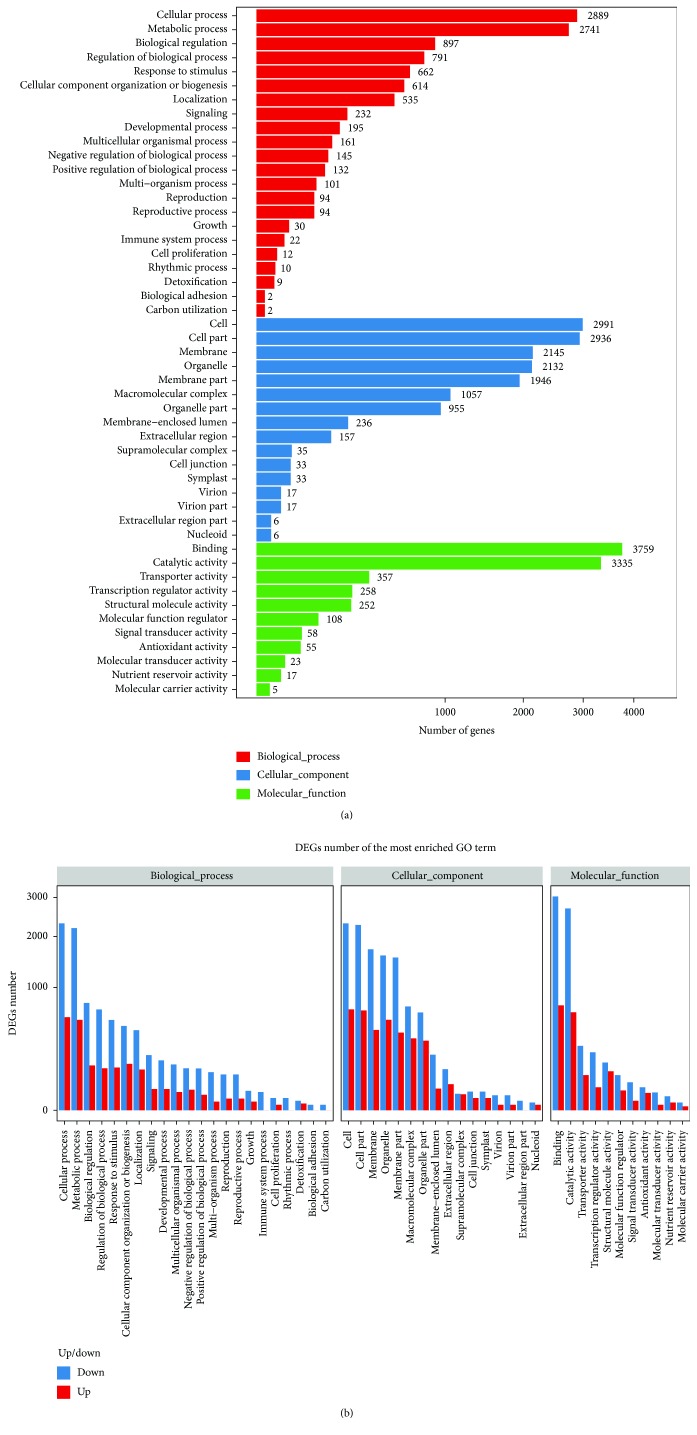
GO analysis of DEGs. (a) GO classification of DEGs. (b) GO classification of upregulated and downregulated genes.

In this research, GO functional classification was performed on 33,299 DEGs. In the cellular component ontology, most DEGs were classified into the cell, cell part, membrane, organelle, membrane part, and macromolecular complex categories. In the molecular function ontology, most DEGs were associated with binding and catalytic activity. In the biological process ontology, most DEGs were classified into the cellular process and metabolic process categories (Figure 4(a)). In every functional category, the downregulated DEGs outnumbered the upregulated DEGs. Additionally, only downregulated DEGs were found in the immune system process, rhythmic process, biological adhesion, carbon utilization, and extracellular region categories (Figure 4(b)).

### 3.5. KEGG Functional Analysis of Differentially Expressed Genes

**Figure 5:**
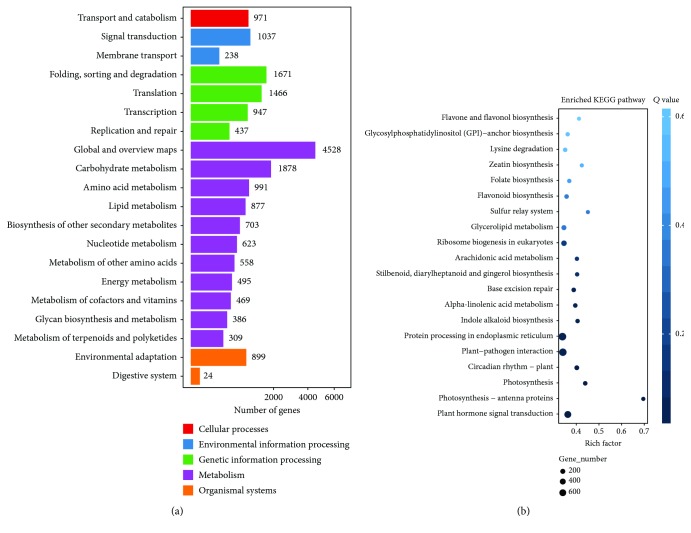
KEGG analysis of DEGs. (a) Pathway classification of DEGs. (b) Pathway functional enrichment result of DEGs.

According to the sequencing results, we classified the DEGs into KEGG biological pathways and conducted enrichment analysis. A total of 19,507 DEGs were annotated in these five pathways. The metabolism pathway contained the most DEGs (11,817 genes, accounting for 60.58%), followed by the genetic information processing pathway (4,521 genes, accounting for 23.18%). The third largest group was the environmental information processing pathway (1,275 DEGs, accounting for 6.54%). Within the metabolism pathway, most DEGs were annotated to “global and overview maps” and “carbohydrate metabolism,” with 4,528 and 1,878 DEGs, respectively. Within the second largest group (genes annotated to genetic information processing), most DEGs were involved in “folding, sorting, and degradation” and “translation,” with 1,671 and 1,466 DEGs, respectively. Within the environmental information processing pathway, 1,037 DEGs were annotated with “signal transduction.” Besides the three major pathways, 971 and 923 DEGs were, respectively, annotated to the cellular processes and organismal system pathways (Figure 5(a)).The DEGs of dormant aerial bulbs and low-temperature-treated aerial bulbs were mostly concentrated in pathways such as protein processing in the endoplasmic reticulum, plant-pathogen interaction, plant hormone signal transduction, and ribosome biogenesis in eukaryotes, with significant enrichment (Figure 5(b)).

### 3.6. Screening of Key Differentially Expressed Genes in Aerial Bulbs Treated with Low Temperature in Calcium and Hormonal Signal Transduction Pathways

We screened out 14 significantly upregulated DEGs participating in calcium signal transduction within aerial bulbs treated with low temperature, including *DPK*, *CML*, *CLB*, *iPLA2*, *CSC1*, *TCTP*, *RALF*, *NDB2*, and *CAM*, the log2 fold change values of which were all above 2. Four genes (*CDPK*, *CLB*, *TCTP*, and *RALF*) were only expressed in aerial bulbs treated with low temperature and not expressed in dormant aerial bulbs, with log values all above 6 (Table 2).

**Figure 6:**
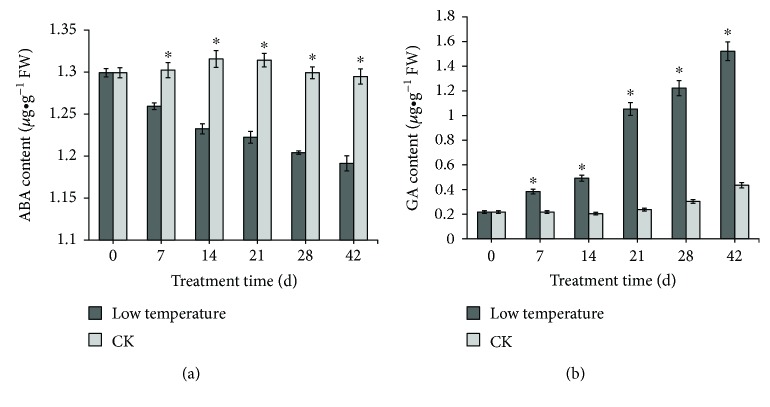
Content of ABA (a) and GA (b) in aerial bulbs treated with low temperature.

Low-temperature treatment significantly affected endogenous hormone contents of aerial bulbs. Compared with levels in untreated bulbs, endogenous gibberellin content increased significantly following treatment, while the content of abscisic acid decreased significantly (Figures 6(a) and 6(b)).

**Figure 7:**
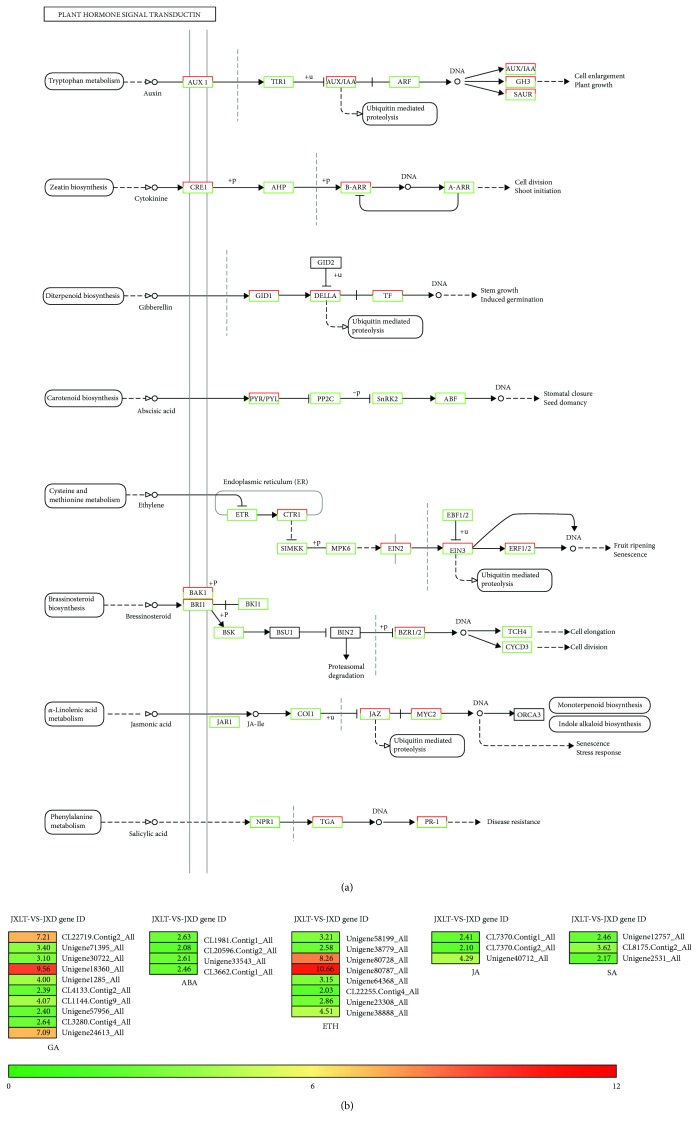
Plant hormone signal transduction and the relative heat map. (a) Plant hormone signal transduction. (b) The heat map of DEGs involved in plant hormone signal transduction.

The hormonal signal transduction diagram is shown in Figure 7(a). Red represents the upregulated gene, and green represents the downregulated gene. There are 14 significantly upregulated DEGs participating in the transduction of GA and ABA hormone signals within aerial bulbs treated with low temperature, including *GAI1*, *GAST1*, *GA2ox*, *GA20ox*, and *GASA* in the GA signaling pathway and *NCED1*, *CYP707*, *RCI2A*, and *PP2C* in the ABA signaling pathway. The *GAI1* and *GA2ox* genes were only expressed in aerial bulbs treated with low temperature and not expressed in dormant aerial bulbs, and the log values of both were above 7 (Table 3). Their expression was consistent with the changes of endogenous hormone content in aerial bulbs. It was speculated that low temperature could activate the biosynthesis and signal transduction of GA by inhibiting the expression of DELLA gene *GAI1*, a negative regulator of the GA signal pathway, so as to release dormancy of aerial bulbs. At the same time, the expression of negative regulatory genes of ABA such as *CYP707* and *PP2C* was significantly upregulated, which resulted in the relative decrease of ABA content, which was conducive to the relief of dormancy. Additionally, there are 14 significantly upregulated DEGs participating in the transduction of ethylene (ETH), jasmonic acid (JA), and salicylic acid (SA) hormone signals within aerial bulbs treated with low temperature, including *EIN2*, *ACS8*, *ACS12*, and *ACO1* in the ETH signaling pathway, *MYC2* in the JA signaling pathway, and *TGA* and *PR-1* in the SA signaling pathway (Table 4).  Figure 7(b) is the relative heat map of DEGs involved in plant hormone signaling pathways screened out in this study.

### 3.7. Identification and Classification of Upregulated Differentially Expressed Transcription Factors in Aerial Bulbs Treated with Low Temperature

**Figure 8:**
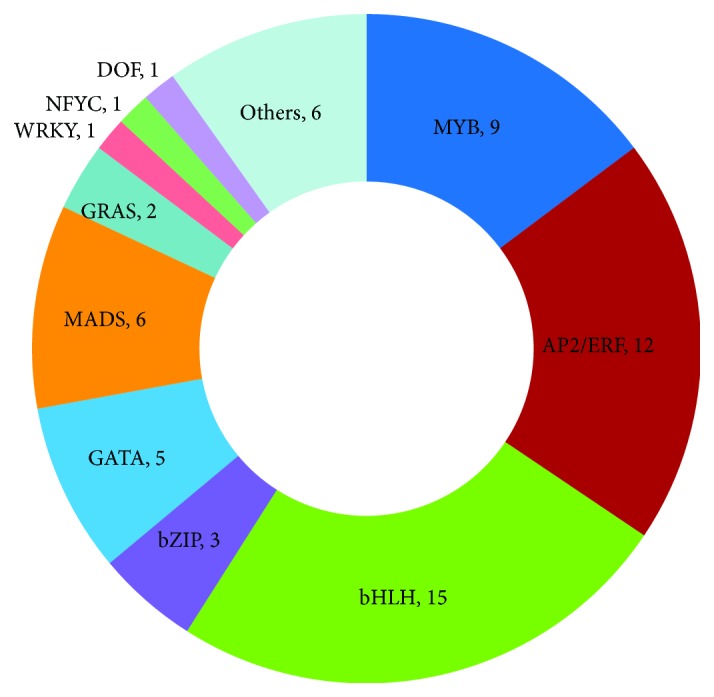
Classification of significantly upregulated TFs.

After low-temperature treatment, the aerial bulbs contained a total of 61 significantly upregulated transcription factors, the log2 fold change values of which were all above 2. These transcription factors were classified into 15 different families, among which the bHLH family contained the most transcription factors (15), followed by the AP2/ERF family (12), the MYB family (9), and the MADS family (6) (Figure 8, Supplementary Table S1). This provides basic information about the roles of transcription factors in breaking the dormancy of garlic aerial bulbs at low temperature.

### 3.8. qRT-PCR Validation of DEGs

**Figure 9:**
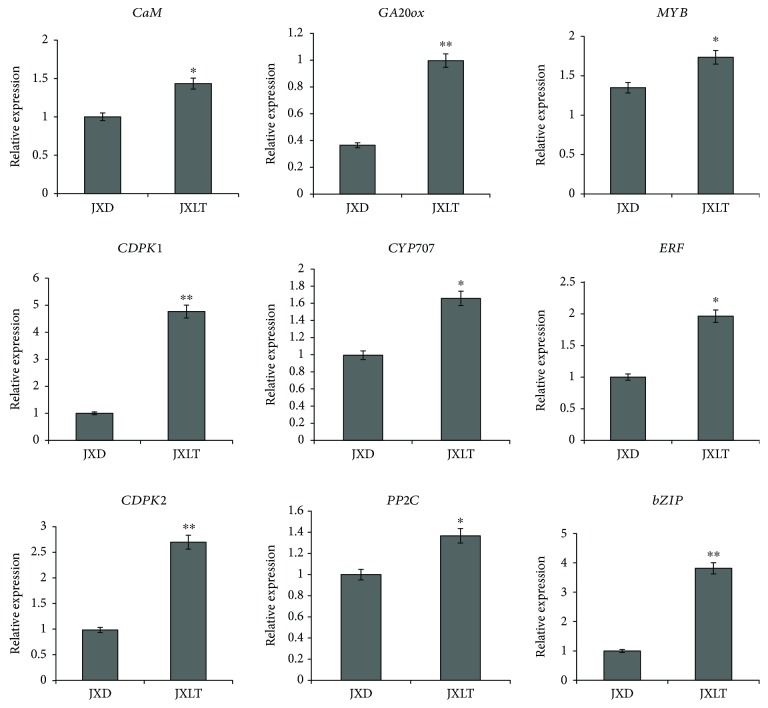
qRT-PCR analysis of 9 key differentially expressed genes. Statistical significance: ^∗^ *P* < 0.05; ^∗∗^ *P* < 0.01.

We selected nine genes from the DEGs and designed primers for them for fluorescence quantitative gene expression analysis (Table 5). The expression levels of the *CaM* and *CDPK* genes in the calcium signaling pathway were significantly higher in aerial bulbs treated with low temperature than in dormant aerial bulbs. The expression levels of the *GA20ox* (a key gene for GA synthesis), *CYP707* (an ABA decomposition gene), and *PP2C* (a negative regulatory factor gene) genes were greatly increased in aerial bulbs treated with low temperature. The expression levels of the *MYB*, *ERF*, and *bZIP* transcription factor genes in aerial bulbs treated with low temperature were also remarkably increased (Figure 9). The above results of fluorescence quantitative analysis were all consistent with the transcriptome sequencing results, indicating that our transcriptome sequencing data were accurate and reliable and could be used as a reference in subsequent studies. It can be concluded that the key DEGs screened out in this experiment are significant for the breaking of aerial bulb dormancy induced by low temperature.

## 4. Discussion

In this study, we used the high-throughput sequencing technique to assemble, transcribe, and analyze the transcriptome of garlic aerial bulbs with no reference genome. We then identified and analyzed the DEGs of aerial bulbs during dormancy and after low-temperature treatment and obtained a total of 43,271 DEGs. Our results confirm that the high-throughput transcriptome sequencing technique is an effective platform for studying organisms with no reference genome. The transcriptome data for garlic aerial bulbs obtained here provide an ideal sequence resource for the development of garlic molecular biology and cell markers in the future. We identified a total of 15,954 Unigenes showing expression differences in excess of two times between dormant and low-temperature-treated aerial bulbs, among which the expression differences of 1,798 Unigenes exceeded five times, and those of 18 Unigenes exceeded 10 times. Additionally, 925 Unigenes were only expressed in aerial bulbs treated with low temperature, and 1,278 Unigenes were only expressed in dormant aerial bulbs, indicating that these Unigenes function in a specific biological period. We observed that 33,299 Unigenes were distributed in multiple GO categories. The genes with upregulated expression after low-temperature treatment were mainly associated with cellular processes, metabolic processes, nucleotide binding, and catalytic activity. These results show that garlic aerial bulbs treated with low temperature are metabolically active and their dormancy is broken.

Ca^2+^ has been shown to be an important regulatory factor during eukaryotic cell division [22]. The level of Ca^2+^ in the cytoplasm is related to the transmission of low-temperature signals in cells [23]. The plant cell membrane senses low temperatures and transmits a low-temperature signal further downstream via Ca^2+^, ABA, and other second messengers, among which the calcium ion is an important second messenger for transducing the low-temperature signal [24]. The major calcium sensors inside plants are calmodulin (CaMs), calcium-dependent protein kinases (CDPKs), calcineurin B-like proteins (CBLs), and CBL-interacting protein kinases (CIPKs), which can transmit information to downstream target genes, induce changes in gene expression, and ultimately cause physiological changes [25]. When cells are stimulated by low temperatures, the intracellular free Ca^2+^ concentration increases, and calcium signaling is activated in combination with calmodulin. Ca^2+^ can then regulate plant growth. In this study, both *CDPK* and *CAM* were significantly upregulated in aerial bulbs treated with low temperature, indicating that low-temperature treatment can induce the generation of calcium signals in aerial bulbs. This result is consistent with the breaking of dormancy.

Overexpression of the key ABA-decomposing enzyme ABA 8′-hydroxylase (*cyp707*) reduces the ABA content in seeds and therefore helps release seeds from dormancy [26]. Gosti et al. found a gene family with phosphatase activity (protein phosphatase 2C (PP2C)) active during the germination of spinach seeds, with deficient mutants sensitive to ABA, which was probably related to the negative regulation of the ABA signaling pathway [27]. In this research, the expression levels of the *CYP707* and *PP2C* genes were both increased in garlic aerial bulbs treated with low temperature, and consequently, the ABA content was decreased. These results are consistent with the breaking of dormancy. The above results indicate that plants can regulate germination and dormancy through ABA. By changing the ABA signaling pathway, it is possible to help release the germination of plant materials from suppression. The role of GA in plant germination is opposite to that of ABA, and it can antagonize ABA's suppression and promote germination. Mutants lacking GA cannot germinate normally, indicating that GA is a major regulatory factor for the breaking of dormancy in garlic [28]. GA20 oxidase (GA20ox) directly catalyzes the formation of biologically active GA and is the key enzyme regulating GA biosynthesis [29]. In garlic aerial bulbs treated with low temperature, the expression level of GA20 was increased and so was that of the GASA gene, the expression of which is regulated by GA. Other genes involved in the biosynthetic metabolism and signal transduction of GA were also upregulated. It can therefore be speculated that the endogenous GA content was increased during the low-temperature-induced breaking of dormancy in aerial bulbs, which may be one of the factors promoting the germination of aerial bulbs. This result is consistent with the breaking of dormancy. The genes discovered in this research related to the metabolism of relevant hormones will be of great importance in determining the composition of hormones added to the culture medium to improve the seedling rate in virus-free garlic seedling breeding.

Transcription factors play an important regulatory role in physiological activities such as growth and morphogenesis in plants. In recent years, many studies have investigated the transcription factors involved in plant germination, such as the *Arabidopsis thaliana* MYBC1 transcription factor involved in ABA-mediated seed germination and the AP2-type transcription factor with specific expression in soybean seeds that participates in seed germination. In this research, we identified 61 transcription factors that were significantly upregulated in aerial bulbs treated with low temperature, including 9 MYB, 6 MADS, 2 GRAS, 12 AP2/ERF, and 1 WRKY transcription factors. MYB transcription factors, as the largest group of plant transcription factors, participate extensively in physiological and biochemical reactions in plants and play an important role in vital plant processes, such as cellular morphology and pattern formation, regulation of secondary metabolism, and biotic and abiotic stress responses. MADS transcription factors are involved in almost every aspect of plant growth and development, such as root growth and development [30, 31], differentiation of apical meristems [32], fruit development [33, 34], fruit maturation and dehiscence [35, 36], regulation of photosynthesis and nutrient metabolism [37], and the transduction of hormonal signals [38]. The plant GRAS protein family plays a key regulatory role in normal growth and stress responses to the environment, but very few studies have so far been conducted on the functions of known GRAS proteins. The AP2/EREBP protein family contains a large number of plant transcription factors that can be divided into three subfamilies, i.e., AP2, EREBP, and RAV 3 [39]. AP2 subfamily proteins mainly participate in plant growth and development, such as by regulating the development of meristems, ovules, and seeds [40–42]. The EREBP subfamily mainly participates in response to plant hormones and biotic and abiotic stresses, such as heat and cold, high salinity, and drought [43, 44]. The RAV subfamily plays an important role in plant responses to ethylene [45], brassinolide [46], and biotic and abiotic stresses [44]. The WRKY family is a new transcription factor family specifically found in plants in recent years that is closely related to growth and development, senescence, and stress responses in plants. WRKY members are involved in ABA signaling pathways and act as negative regulatory factors. We found one WRKY transcription factor in aerial bulbs according to our gene function annotation. Its expression level was significantly upregulated, indicating that the synthesis of endogenous ABA was decreased during the germination of aerial bulbs at low temperature. A decrease in ABA may be conducive to the germination of aerial bulbs.

**Figure 10:**
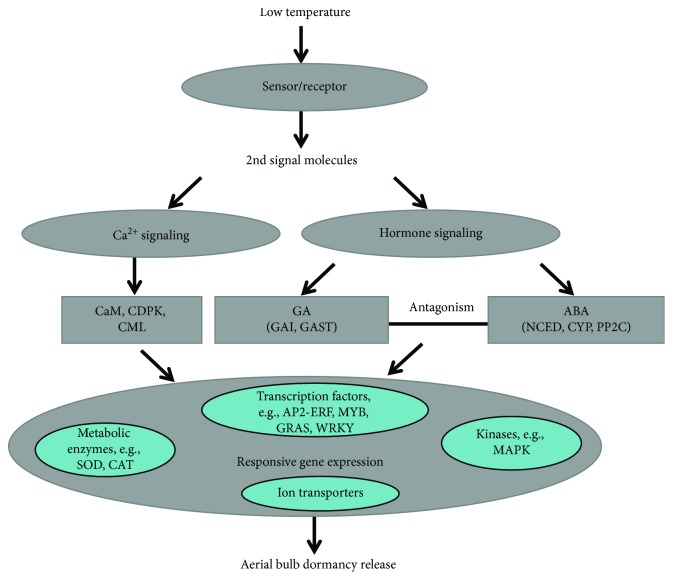
The regulatory mechanism of dormancy release during low-temperature treatment of aerial bulbs.

Low temperature is an important environmental signal for breaking dormancy. Based on our transcriptome sequencing results, we have constructed a complex dormancy-regulation network that includes the many DEGs we discovered (Figure 10). After low-temperature treatment, biological processes such as transcription regulation and plant hormone and calcium signal transduction are activated. These biological changes in aerial bulbs may be related to the low-temperature-induced breaking of dormancy. This research provides an important basis for further understanding the dormancy mechanism in garlic aerial bulbs and also supplements the molecular biological data available for garlic.

## 5. Conclusions

This research shows that high-throughput transcriptome sequencing provides an effective method for identifying the key genes involved in the low-temperature-induced breaking of dormancy in garlic aerial bulbs without a reference genome. We preliminarily screened out genes related to the breaking of dormancy in garlic aerial bulbs after low-temperature treatment, including genes related to calcium signaling, hormonal signaling, and transcription factors. We explored the relationship between the DEGs and the low-temperature-induced breaking of dormancy in aerial bulbs. Our results provide a theoretical basis for breaking the dormancy of aerial bulbs with low-temperature treatment to produce virus-free seedlings and increase the output and quality of garlic.

## Figures and Tables

**Table 1:** Summary of sequence analysis.

Sample	Raw reads (Mb)	Clean reads (Mb)	Clean bases (Gb)	Q20 (%)	Q30 (%)	GC content (%)
JXD1	67.71	65.22	6.52	97.22	89.18	39.36
JXD2	69.90	66.68	6.67	96.67	87.98	39.45
JXD3	67.53	65.49	6.55	97.16	88.89	39.13
JXLT1	69.57	67.40	6.74	96.49	87.44	39.77
JXLT2	69.69	67.42	6.74	97.05	88.63	40.34
JXLT3	67.36	65.33	6.53	97.14	88.95	40.12
JXD summary	205.14	197.39	19.74	97.02	88.68	39.31
JXLT summary	206.62	200.15	20.01	96.89	88.34	40.08
Summary	411.76	397.54	39.75	96.96	88.51	39.70

**Table 2:** Key differentially expressed genes involved in calcium signal transduction.

Unigene	Gene name	Annotation	FPKM (JXD)	FPKM (JXLT)	Log2 fold change (JXLT/JXD)
Unigene58778_All	CDPK	Calcium-dependent protein kinase	66	354	2.29
CL7012.Contig3_All	CDPK	Calcium-dependent protein kinase	17.75	142.93	2.88
CL3235.Contig4_All	CDPK	Calcium-dependent protein kinase	10.51	55.33	2.26
CL3235.Contig3_All	CDPK	Calcium-dependent protein kinase	0	57.28	6.71
CL4941.Contig1_All	CML	Calcium-binding protein	289.43	1273.42	2.01
CL4132.Contig9_All	CLB	Ca^2+^-dependent lipid-binding protein	0	128.97	7.88
CL5045.Contig5_All	iPLA2	Ca^2+^-independent phospholipase A2	78.92	359.51	2.06
Unigene45967_All	iPLA2	Ca^2+^-independent phospholipase A2	8	112	3.68
Unigene15249_All	CSC1	Calcium permeable stress-gated cation channel	74.83	424.91	2.37
Unigene25677_All	CSC1	Calcium permeable stress-gated cation channel	15.56	92.51	2.44
Unigene82102_All	TCTP	Translationally controlled tumor protein	0	62	6.82
CL18975.Contig2_All	RALF	Rapid alkalinization factor	0	111	7.66
CL783.Contig4_All	NDB2	NAD(P)H-ubiquinone oxidoreductase B2	197.68	1178.62	2.44
Unigene69223_All	CaM	Calmodulin	171	865	2.21

**Table 3:** Key differentially expressed genes involved in hormone signal transduction of GA and ABA.

Unigene	Gene name	Annotation	FPKM (JXD)	FPKM (JXLT)	Log2 fold change (JXLT/JXD)
Unigene30722_All	GAI1	Gibberellin response modulator	42.21	395.12	3.10
Unigene18360_All	GAI1	Gibberellin response modulator	0	413.97	9.56
CL22719.Contig2_All	GAST1	GA-stimulated transcript	1	161.91	7.21
CL4133.Contig2_All	GAST1	GA-stimulated transcript	23.33	133.58	2.39
Unigene71395_All	GA2ox	Gibberellin 2-oxidase	22	255	3.40
CL3280.Contig4_All	GA2ox	Gibberellin 2-oxidase	17.01	115.74	2.64
Unigene24613_All	GA2ox	Gibberellin 2-oxidase	0	74.42	7.09
Unigene57956_All	GA20ox	Gibberellin 20-oxidase	10	58	2.40
Unigene1285_All	GASA3	Gibberellin-regulated protein 3	66	1153	4.00
CL1144.Contig9_All	GASA6	Gibberellin-regulated protein 6	6.1	112.6	4.07
Unigene33543_All	NCED1	9-*cis*-Epoxycarotenoid dioxygenase 1	17	114	2.61
CL1981.Contig1_All	CYP707	Abscisic acid 8′-hydroxylase	51	346	2.63
CL20596.Contig2_All	RCI2A	Hydrophobic protein	98.5	456.22	2.08
CL3662.Contig1_All	PP2C	Protein phosphatase 2C	25.54	154.06	2.46

**Table 4:** Key differentially expressed genes involved in signal transduction of other hormones.

Unigene	Gene name	Annotation	FPKM (JXD)	FPKM (JXLT)	Log2 fold change (JXLT/JXD)
Unigene58199_All	EIN2	Ethylene-insensitive 2	14	142	3.21
Unigene38779_All	ACS8	1-Aminocyclopropane-1-carboxylate synthase 8	9	59	2.58
Unigene80728_All	ACS12	1-Aminocyclopropane-1-carboxylate synthase 12	0	168	8.26
Unigene80787_All	ACS12	1-Aminocyclopropane-1-carboxylate synthase 12	0	887	10.66
Unigene64368_All	ACS	1-Aminocyclopropane-1-carboxylate synthase	8	78	3.15
CL22255.Contig4_All	ACO1	Aconitate hydratase 1	38.8	174.17	2.03
Unigene23308_All	ACO1	1-Aminocyclopropane-1-carboxylate oxidase 1	31.64	252.36	2.86
Unigene38888_All	ACO1	1-Aminocyclopropane-1-carboxylate oxidase 1	5.2	129.47	4.51
CL7370.Contig1_All	MYC2	Transcription factor MYC2	163	949	2.41
CL7370.Contig2_All	MYC2	Transcription factor MYC2	235	1101	2.10
Unigene40712_All	MYC2	Transcription factor MYC2	4	86	4.29
Unigene12757_All	TGA	Transcription factor TGA	31.7	191.05	2.46
CL8175.Contig2_All	TGA	Transcription factor TGA	33.11	445.91	3.62
Unigene2531_All	PR-1	Pathogenesis-related protein	13	64	2.17

**Table 5:** Differentially expressed genes and their primer sequences in qRT-PCR.

Unigene	Gene name	Annotation	Primer sequence (5′ → 3′)
Unigene69223_All	CaM	Calmodulin	YF: GACAAGGATCAGAATGGTTAC YR: TTCACTTAGCCAACATCATC

CL3235.Contig4_All	CDPK1	Calcium-dependent protein kinase	YF: TGTCCTCTTCCAACTTCTT YR: CCAGCTAAGCACATCAAG

Unigene58778_All	CDPK2	Calcium-dependent protein kinase	YF: GCCATACCACTATTGACTTG YR: CTCCACTTCTACCGATTGA

Unigene57956_All	GA20ox	Gibberellin oxidase	YF: TCTCGCAAACTCATTAACAG YR: GCATAGCCTTCCATCTGA

CL1981.Contig1_All	CYP707	ABA 8′-hydroxylase	YF: CAGGCATTGGATTAGCATAA YR: ACTGAGGTAGGTATGTGAAC

CL3662.Contig1_All	PP2C	Protein phosphatase 2C	YF: AGTGCTGCAAGTAGACAT YR: CGCTCCTGTTATAGGTTTC

Unigene68397_All	MYB	MYB transcription factors	YF: GGAGATTGGCAATGAAGG YR: ATCACCTAACGAACTTCCT

CL11702.Contig2_All	ERF	AP2/ERF transcription factors	YF: AGAGGAGGTGGAGATATGA YR: GAGATGGAGACGATAGAAGAA

Unigene50859_All	bZIP	bZIP transcription factors	YF: AGGAAGTTGGTTCTCTGATT YR: GATGCCCTTCATGTAAAGTT

	Actin	Reference gene	YF: TCCTAACCGAGCGAGGCTACAT YR: GGAAAAGCACTTCTGGGCACC

## Data Availability

The RNA-Seq data used to support the findings of this study have been deposited in the Short Read Archive at the National Center for Biotechnology Information.
